# Changes in Circulating Lysyl Oxidase-Like-2 (LOXL2) Levels, HOMA, and Fibrosis after Sustained Virological Response by Direct Antiviral Therapy

**DOI:** 10.3390/jcm8081242

**Published:** 2019-08-17

**Authors:** Angela Puente, Jose Ignacio Fortea, Miguel Posadas, Agustin Garcia Blanco, Laura Rasines, Joaquin Cabezas, Maria Teresa Arias Loste, Susana Llerena, Paula Iruzubieta, Emilio Fábrega, Javier Crespo

**Affiliations:** 1 Digestive Disease Department, Marqués de Valdecilla University Hospital, 39008 Santander, Spain; 2 Health Research Institute Marques de Valdecilla, IDIVAL, 39011 Santander, Spain; 3 Medicine and Psychiatry Department, Cantabria University, 39011 Santander, Spain

**Keywords:** SVR, liver fibrosis, LOXL2, portal hypertension, fibrosis regression

## Abstract

Background: we aimed to assess the influence of metabolic syndrome on fibrosis regression (using liver-stiffness measurement (LSM) and serological scores) and the relationship with the expression of lysyl oxidase-like-2 as a potential goal of antifibrotic therapy. Methods: We included 271 patients treated with Direct Antiviral Therapy (DAAs) in our hospital who achieved a sustained virological response (SVR); physical examination, blood tests, and LSM were made at baseline (B) and 24 months (24 M) after SVR. Hemodynamic studies and transjugular liver biopsies were performed on 13 patients. Results: At B, 68 patients were F1 (25.1%); F2 *n* = 59 (21.7%); F3 *n* = 44 (16.05%); and 100 were F4 (36.9%). Although the LSM (absolute value) improved in 82% of patients (*n* = 222), it progressed in 17.5% of patients (*n* = 48). At 24 M, 48 patients met the metabolic syndrome (MetS) criteria and there was an increase in patients with a BMI of >25 kg/m^2^ (*p* < 0.001). At B and 24 M, a BMI of >25 kg/m^2^ is a risk factor for significant fibrosis or steatosis at 24 M (*p* < 0.05) and progression on LSM (*p* < 0.001), as well as MetS at B and 24 M (OR 4.1 IC (1.4–11.7), *p* = 0.008; and OR 5.4 IC (1.9–15.4), *p* = 0.001, respectively). Regarding the correlation between LSM and the liver biopsy, we found that only six out of 13 patients had a matching LSM and biopsy. We found a statistically significant decrease in LOXL2 levels at 24 M with respect to B (*p* < 0.001) with higher serological value in patients with elastography of >9 kPa vs. <9 kPa (*p* = 0.046). Conclusion: Regression of LSM was reached in 82% of patients. Downregulated LOXL2 was demonstrated post-SVR, with overexpression in cirrhotic patients being a potential therapy goal in selected patients.

## 1. Introduction

Patients with chronic hepatitis C virus have experienced radical changes in the evolution of their disease after the appearance of new direct-acting antivirals, achieving virological healing rates of over 95%, even in very advanced phases of the disease [1]. Achievement of the sustained virological response (SVR) showed that it slows down disease evolution, slowing the appearance of esophageal varices, clinical decompensation, or hepatocarcinoma, and even achieving the regression of hepatic fibrosis [2,3,4,5].

However, we know that disease regression is not a universal fact. Previous studies have shown that up to 15% of patients with chronic HCV liver disease do not achieve fibrosis regression (as determined by elastographic methods), and this fact can occur both in the advanced and earlier stages [6]. This suggests that regression not only depends on the so-called point of no return (thickened fibrotic septa and clinically significant portal hypertension), but there may also be cofactors (such as metabolic syndrome or alcohol) that can be key in this progression and they are often not sufficiently evaluated in this patient group [2,7,8]

The fibrogenesis process, which is common to all forms of liver injury, is characterized by increased extracellular matrix (ECM) components, including collagen deposition (collagen types I > III > IV), proteoglycans, and glycoproteins (laminin and fibronectin 2) [9,10,11]. Stellate and Kupffer cells (liver macrophages) are the key element in fibrogenesis, and the activation of Ly6Chigh macrophages is the basis for regression [12,13,14].

The focus on irreversible biochemical changes has allowed for the identification of lysyl oxidase 2 (LOXL2), an enzyme that promotes the network of collagen fibers of the extracellular matrix [15,16]. LOXL2 is a Cu-dependent amine oxidase capable of post-transcriptional modification of type 1 collagen (the main collagen in hepatic fibrosis) and elastin by oxidation of peptidyl lysine and hydroxylysine residue collagen, transforming it into allysine, a responsible crosslink formation that stabilizes collagen and elastin in the extracellular matrix. LOXL2 upregulation was detected during insulin resistence (IR) induced by obesity, and NAFLD patients with type 2 diabetes [16,17,18].

To date, no correlation has been demonstrated in the serum levels of LOXL2 and liver expression in SVR patients, but the relationship between LOXL2 serum levels and elastography, as well as hepatic venous pressure in cirrhosis due to nonalcoholic steatohepatitis (NASH) and hepatitis C, has previously been communicated, [19,20,21]. The use of the monoclonal antibody Simtuzumab like an antifibrogenic drug is in question [22,23,24,25,26].

Another issue to be taken into account in the method is the validity of elastography (mainly Fibroscan^®^) and other serological methods in the follow-up of post-SVR fibrosis and steatosis [27]. Liver-stiffness measurement (LSM) by Fibroscan^®^ improved after viral elimination due to the drop in necroinflammatory activity, but it is not a good screening method of clinically significant portal hypertension since it was recently shown that at 24 and 96 weeks after treatment, 43% and 28% of patients with a LSM of <13.6 kPa have a hepatic venous pressure gradient (HVPG) of >10 mmHg [27,28,29]. The accuracy of controlled attenuation parameters (CAP) in steatosis screening in that cohort of patients needs to be confirmed.

We aimed to assess the influence of metabolic syndrome in fibrosis regression (by liver-stiffness measurement and serological scores) and its relationship with the expression of lysyl oxidase-like-2 as a potential goal of antifibrotic therapy.

## 2. Patients and Methods

Patients: This was a cohort study conducted at the Marqués de Valdecilla University Hospital with 2 retrospective and prospective phases.

Inclusion criteria: Patients (1) treated with the new direct-action antivirals at Marqués de Valdecilla University Hospital from October 2014 to May 2016, independently of a pretreatment liver-fibrosis stage (determined by LSM); and (2) aged between 18 and 85 years. 

Exclusion criteria: (1) Coexistence of HIV/HBV coinfection or autoimmune hepatitis, (2) hepatocarcinoma, (3) any comorbidity that entails a therapeutic limitation and/or a prognosis of life less than 12 months, (4) pregnancy or lactation, (5) TIPS or portosystemic shunt, and (6) participation in a clinical trial.

### Methods

All patients included in the study had a complete physical examination at baseline and 24 months (24 M), including blood tests with biochemistry, liver function, insulin, blood count, and coagulation, and a Fibroscan^®^. Blood and plasma samples were stored in the IDIVAL Biobank. We analyzed the data stored in our database and in the electronic health record of the Cantabrian Health Service related to patients included in the study. Baseline cirrhotic patients and progressors at 24 M were considered for liver hemodynamic and transjugular liver biopsy.

All included patients signed informed consent approved by the local ethics committee of the Marqués de Valdecilla University Hospital. This study was also conducted according to the principles expressed in the Declaration of Helsinki. 

Definitions, procedures, and statistical analysis are described in Supplementary File S1. 

## 3. Results

### 3.1. Study Population

Between October 2014 and May 2016, 308 patients with HCV-related liver disease were treated with all-oral antiviral therapy in the Marqués de Valdecilla University Hospital. Among the 308 initially enrolled patients, 279 accepted inclusion in the study, five of whom did not have baseline LSM, so they were not included. The total number of included patients was 271.

Baseline characteristic of the patients are shown in Table 1. 

#### Baseline Characteristics and Impact of SVR at Two Years

**Figure 1:**
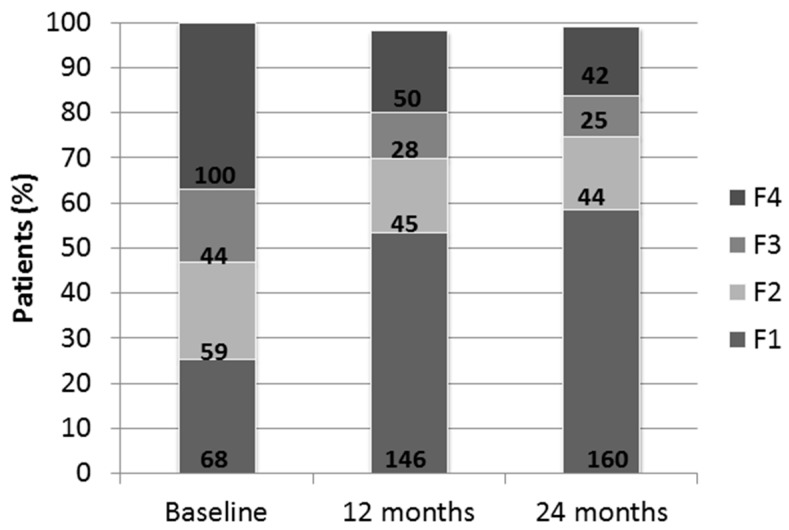
Distribution-elastography data by Fibroscan ^®^ in 271 patients at baseline and 12 and 24 months. Distribution of patient percentage in order of elastography classification. Bold numbers indicate absolute values.

At 24 M, 160 patients were F1 (59%); 44 patients were F2 (16.2%); 25 patients were F3 (9.2%), and 42 patients were F4 (15.4%) (Figure 1).

Although LSM (absolute value) improved in 82% of patients (*n* = 222), it progressed in 48 patients (17.5%) and remained stable in one patient (0.36%). The mean value of LSM improved from 12.76 ± 1.7 kPa at baseline to 8.22 ± 6.32 kPa at 24 M (*p* < 0.001), and a non-significant increase of CAP (227.50 ± 84.56 dB/m vs. 229.29 ± 60.98 dB/m; *p* = 0.916) was found. However, these data must be cautiously taken because we only had 25 cases with baseline CAP. At 24 M, 67 patients had significant LSM (24.7%) and 17 also had severe steatosis by CAP (6.2%). Furthermore, 42 patients had severe steatosis (CAP > 288 dB/m) without advanced fibrosis.

As expected, improvement on necroinflammatory and liver function were confirmed (ALT, AST, GGT, bilirubin, albumin, and protrombin activity, *p* < 0.001).

At two years, five more patients (*n* = 48) met the criteria of metabolic syndrome (MetS). We detected an increase of patients with BMI ≥ 25 kg/m^2^: 58.8% vs. 59.5% (*p* < 0.001) in relation to the baseline. However, we did not find a significant worsening of BMI compared with the baseline (26.5 ± 4.21 vs. 27.36 ± 11.8 kg/m^2^; *p* = 0.221). 

The metabolic biochemical components worsened at 24 M compared to baseline: triglycerides 99.43 ± 45.71 vs. 109 ± 56.04 mg/dL, *p* = 0.012; total cholesterol 166.31 ± 38.04 vs. 189 ± 40.9 mg/dL, *p* = 0.001; and LDL-cholesterol 96.52 ± 30.5 vs. 119.39 ± 29.702 mg/dL, *p* = 0.001, while HOMA mildly improved (*p* = NS). We found that insulin (*r* = 0.356, *p* < 0.0001), HOMA (*r* = 0.385, *p* < 0.0001), and MetS OR 3.4 (0.700; 6.294; *p* < 0.001) were the most important factors of advanced fibrosis by Fibroscan^®^ in univariate logistic regression analysis.

### 3.2. Noninvasive Serological Scores

Likewise, we studied the usefulness of other noninvasive serological scores for the screening of significant fibrosis at 24 M compared to LSM. We found weak negative correlation with APRI (*r* = –0.165, *p* < 0.006), FORNs (*r* = –0.287, *p* < 0.0001), and FIB 4 (*r* = –0.093, *p* = 0.128), and positive correlation with HEPAMET (*r* = 0.508, *p* < 0.001).

### 3.3. Influence of Metabolic Syndrome in Fibrosis Regression

We found a significant relationship between metabolic variables and noninvasive fibrosis scores at 24 M, as is described in Table 2. At baseline and 24 M, BMI ≥ 25 kg/m^2^ was a risk factor for significant fibrosis or steatosis (>9 kPa or CAP > 288 dB/m) at 24 M (*p* < 0.05), as well as baseline and 24M MetS: OR 4.1 IC (1.4–11.7), *p* = 0.008; and OR 5.4 IC (1.9–15.4), *p* = 0.001, respectively. Progression of liver stiffness and BMI ≥ 25 kg/m^2^ were related (*p* < 0.001, chi-squared).

Univariate analysis showed significant logistic correlation between the values of insulin, LDL cholesterol, HOMA, and APRI. In multivariable analysis; the only one that maintained the significance was the HOMA: *r* = 2.090, *p* =0.025 (Table 2). 

### 3.4. Cirrhotic Patients

A subgroup of 12 patients with pretreatment cirrhosis (LSM > 10.5 kPa) and a case with post-treatment elastographic progression agreed to perform hepatic venous pressure gradient measurement and transjugular liver biopsy. No patient had previous liver decompensation, varices, or hepatocarcinoma. No patient had clinically significant portal hypertension with mean HVPG 3.7 ± 1.14 mmHg.

**Figure 2:**
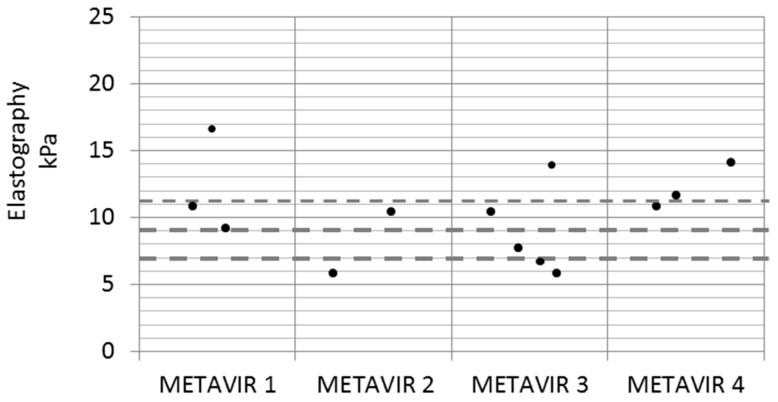
Relationship between Fibroscan^®^ and liver biopsy. We did not find good concordance in the classification of fibrosis between elastography and liver biopsy. Dashed lines indicate elastography cut-offs: 7, 9, and 11 kPa. Number of patients: Metavir 1, *n* = 3; Metavir 2, *n* = 2; Metavir 3, *n* = 4; Metavir 4, *n* = 3.

Regarding the correlation between LSM and liver biopsy, we found that there was a great disparity of values, as shown in Figure 2. No patient with F1 in the liver biopsy had a LSM of <7 kPa, and there were four patients with LSM > 9 kPa that did not have a compatible biopsy (Metavir 1 and 2). Likewise, two patients with LSM < 9 kPa had a histology with bridging fibrosis. Only six patients had matching LSM and biopsy. Interestingly, three patients had a compatible biopsy with NASH with advanced fibrosis, all of them with MetS.

### 3.5. LOXL2 Serum and Tissue Expression

**Figure 3:**
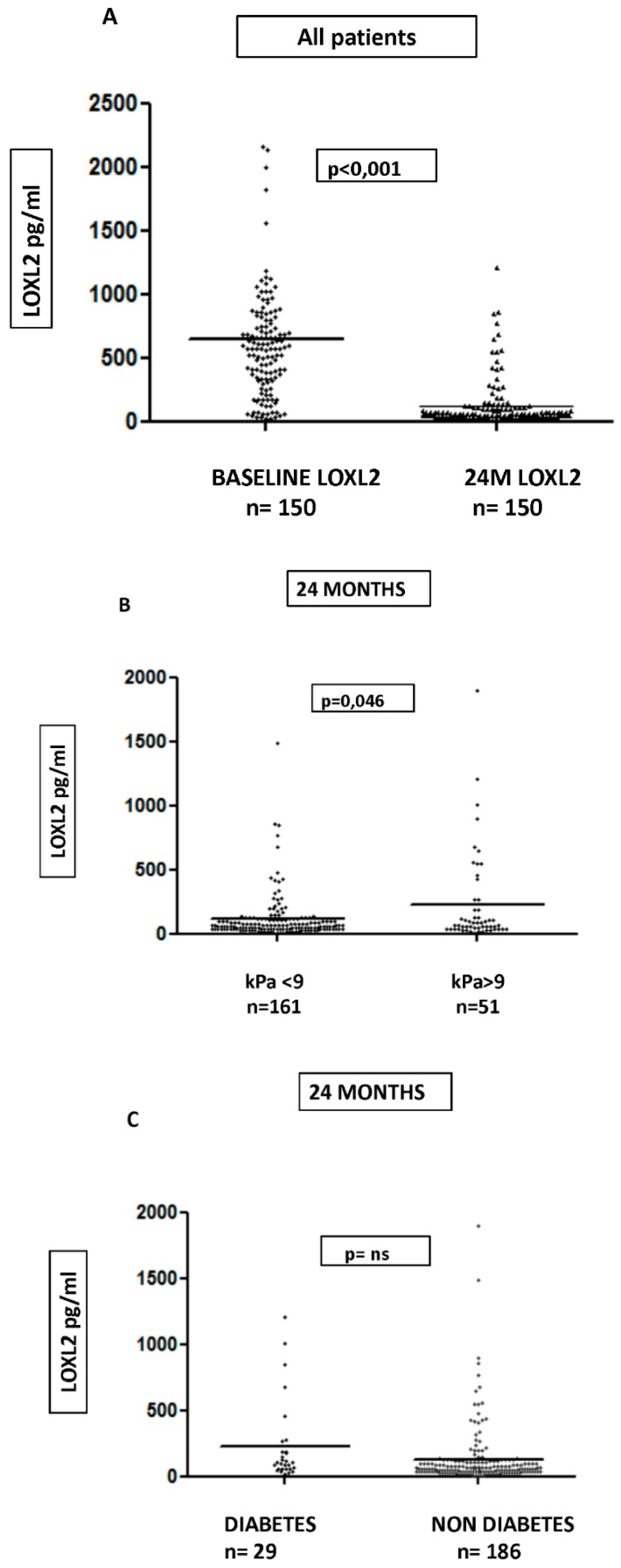
Differences in serum LOXL2 concentration. **A**: *n*= 150, baseline at 891.80 ± 1616 pg/mL vs. 24 M at 328.07 ± 1415.03 pg/mL; *p* < 0.001. **B**: *n* = 212; non-significant fibrosis (kPa < 9) at 113.29 ± 176.96 pg/mL vs. significant fibrosis (kPa > 9) at 216.63 ± 347.92 pg/mL, *p* = 0.046, at 24 months. **C**: *n* = 215 diabetic patients, 231.30 ± 310.28 pg/mL vs. nondiabetics, 127.05 ± 224.15, *p* = 0.09.

We sampled for the measurement of LOXL2 in 150 patients at baseline and in the complete cohort at two years. We found a statistically significant decrease in LOXL2 levels at 24 M in relation to baseline: 891.80 ± 1616 vs. 328.07 ± 1415.03 pg/mL, *p* < 0.001 (Figure 3A)**.** LOXL2 levels were basally correlated with only a HEPAMET score of *r* = 0.208, *p* = 0.032, but not with elastography and other scores.

However, we found great variability in LOXL2 serological values, both at baseline and at 24 M, so, given that the levels of LOXL2 may be increased in other chronic pathologies, we subselected the group of patients without relevant comorbidity (chronic renal insufficiency, kidney transplant, psoriasis, or rheumatological diseases). In the remaining 139 patients, a significant decrease in LOXL2 value (646.51 ± 148.43 vs. 122.03 ± 190.30 pg/mL, *p* <0.001) was maintained.

At 24 M, we found higher serological LOXL2 values in the 212 remaining subjects, with elastography of >9 kPa non-significant fibrosis (216.63 ± 347.92 vs. 113.29 ± 176.96 pg/mL, *p* = 0.046; Figure 3B). 

At baseline, LOXL2 values were associated with platelet count (*r* = 0.228, p0.007; fasting sugar = 0.215, *p* < 0.011) but not with any of the serum scores including elastography (*r* = 0.33, *p* = 0.698). At two years, LSM and LOXL2 had light correlation (*r* = 0.245, *p* = 0.001) with LSM, but it did not allow us to classify according to fibrosis degree. The relationship with platelet count at 24 M stayed at *r* = 0.149, *p* = 0.030. As previously suggested, there is a certain relationship between insulin resistance and LOXL2. In our cohort of patients, we found that diabetic patients had LOXL2 values almost twice that of nondiabetics, although this was not significant (Figure 3C).

The subgroup of 42 cirrhotic patients at 24 M and those in which the LSM progressed had a serum expression three times greater than the group in which LSM improved (405.43 ± 575.92 vs. 161.72 ± 260.4 pg/mL, *p* = 0.09), but this difference was not met in those who did not have advanced fibrosis after treatment (111.64 ± 186 pg/mL [*n* = 22] vs. 113.16 ± 175.59 pg/mL [*n* = 140], *p* = 0.951).

**Figure 4:**
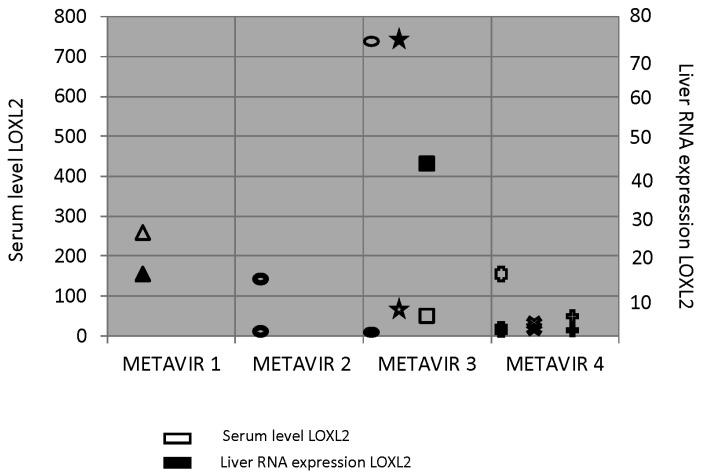
Serum and tissue expression of LOXL2 in eight patients. Each symbol represents a case. Filled symbols represent liver LOXL2 RNA expression, and unfilled symbols represent LOXL2 serum level.

Regarding liver-tissue expression in liver tissue, we obtained a sample valid for analysis from eight patients compared to controls (two healthy liver samples from immunology service Biobank) and found high tissue expression in most patients that was related to serum expression (Figure 4). Again, patients with higher expression of LOXL2 had steatosis and ballooning in the liver biopsy.

## 4. Discussion

The medium- and long-term effect of achieved SVR on liver fibrosis in patients with HCV liver disease is currently a hot topic in hepatology. Recently, evidence suggests a MELD score of ≥18 and the presence of significant clinical portal hypertension as two points of no return in advanced liver disease (including decompensated cirrhosis) [2,5]; more importantly, comorbidities evaluated in a combined liver score (HepCom score) enable detection of a group at high risk of mortality in one or two years, and relevant clinical events after SVR [7].

Another issue to take into account is that although, in most cases, SVR achieves improvement in fibrosis, it is not a negligible percentage (between 24% and 15%) [6] in which advanced fibrosis post-treatment remains. In our study with 271 patients, LSM absolute values improved at two years in 82% of patients (*n* = 222), and progressed in 48 patients (17.5%). If we focus on the subgroup of cirrhotic patients (basally 102 patients), LSM improvement was achieved in 91 patients, and only progressed in 9 patients (ΔLSM: 10.09 ± 12.15 kPa). Fast improvement in LSM should be taken cautiously because the decrease of necroinflammatory activity could misclassify the stage of fibrosis in some patients who have nodular architecture, and screening for liver decompensation is needed [27]. Although we found weak correlation with other noninvasive scores, such as APRI, FORNs, and FIB 4, the utility of these in the post-SVR stage could not be confirmed. In the small subgroup of cirrhotic patients on which hemodynamic and liver biopsy were performed, we found that there was a great disparity of values between LSM and liver biopsy.

In this subset of patients, cofactors such as liver steatosis or MetS could be understood as the driving forces to maintain or even progress liver fibrosis [30,31]. Cardiovascular-risk factors are constant in hepatitis C related to liver disease, and we could observe that, in our cohort of patients, 13.1% are diabetics (of whom 5.4% are insulin-dependent) and 48 met metabolic-syndrome criteria at two years [32]. At 24 M in our cohort, serum triglycerides increased, as well as total cholesterol. This fact is explained by alteration in the lipid metabolism caused by HCV [32]. Despite finding a more favorable lipid profile during the viremic phase, cardiovascular risk was higher than in the general population and emerged after eradication. On the other hand, although insulin resistance improved, this was not significant, and 49 patients (18.1%) had a HOMA of >4 at 24 M compared to 48 at baseline (*p* = NS). In the same way as in the study by Hedenstierna et al., metabolic cofactors such as BMI, HOMA, and MetS were significantly associated with a significant LSM of ≥9 kPa during follow-up. However, with none of these factors distinguished from body mass index, we were unable to predict the worsening of LSM [8].

In this study, we also studied the role of LOXL2 in the regression of post-SVR fibrosis, even though recently published human trials do not support the use of monoclonal antibody Simtuzumab in nonalcoholic steatohepatitis and primary sclerosing cholangitis [24,25,26]. Clearly, LOXL2 serum levels decrease after viral eradication, but we found great variability in values among patients in the cohort that did not allow consistent results. Considering that the LOXL2 pathway could be involved in several oncological, inflammatory, or pulmonary fibrosis diseases, it is not surprising that we found such variability [15,16,17]. After a selection of patients without significant inflammatory comorbidities, results in 150 patients showed significant decrease and a relation with elastography (*r* = 0.245, *p* < 0.0001), and higher serum value was also found in the subgroup of cirrhotic patients with an increase of elastography (*p* = 0.09), but we could not classify different degrees of fibrosis according to LOXL2 serum expression. As previously suggested, there is a certain relationship between insulin resistance and LOXL2. In our cohort of patients, we found that diabetic patients have a LOXL2 value that is almost twice that of nondiabetics, although it is not significant.

To the best of our knowledge, this is the first study that has tried to establish the relationship between serum and tissue expression of LOXL2, and stage of fibrosis. Previous research [20,21] studied changes in liver stiffness and portal pressure gradient but not liver fibrosis by biopsy. Only in eight patients could we demonstrate LOXL2 expression. We found higher tissue expression in most patients and it was related to serum expression but not with liver fibrosis. Again, patients with higher expression of LOXL2 had steatosis and ballooning in the liver biopsy. The provided results can be a starting point when new trials with Simtuzumab are proposed, since correct selection of patients with LOXL2 serum or tissue overexpression would have to be perfect to achieve fibrosis regression. In patients in whom LOXL2 was not shown to be upregulated, we have to look for other liver-fibrosis pathways.

A limitation of our study is that CAP technology was not available in our hospital at the time of study initiation, so we were unable to estimate baseline steatosis prevalence. Another point is the small number of hepatic biopsies performed to date; a larger number would allow us more conclusive results, but undoubtedly, it is an exploratory result that supports the progress of future studies. In addition, quantification of expression by immunohistochemistry, as well as the location of LOXL in liver tissue, would provide more information about the activity of LOXL in fibrosis regression.

Therefore, our study is, according to the best of our knowledge, the one with the largest sample size with a follow-up of two years. In 82% of patients, elastography regression was achieved. Nevertheless, progression of LSM values is not, in the medium term, influenced by MetS or alcohol consumption but by the BMI. In addition, Fibroscan^®^ unreliability in the evaluation of fibrosis in patients with advanced fibrosis before and after treatment was verified, so this must be a topic of extreme interest in the future of hepatology due to the surveillance requirement (mainly hepatocarcinoma screening). Downregulated LOXL2 was almost demonstrated post-SVR, with overexpression in cirrhotic patients being a potential therapy goal in selected patients.

## Figures and Tables

**Table 1:** Baseline demographics and clinical characteristics. Data are presented as no. (%) or mean ± standard deviation. Abbreviations: BMI, body mass index; ALT, alanine aminotransferase; AST, aspartate aminotransferase; GGT, gamma glutamyl transferase; HCC, hepatocellular carcinoma; INR, international normalized ratio.

Variable	*n*/Mean
Demographic Factors (*n* = 271)	
Age (years)	59.29 ± 10.5
Sex	183 (66.8%) men / 91 (33.2%) woman
BMI (kg/m^2^)	Overall = 26.48 ± 4.21 − Normal (<25 kg/m^2^) *n* = 114 (41.6%) − Overweight (≥25 to <30 kg/m^2^) *n* = 109 (39.8%) − Obese (≥30 kg/m^2^) *n* = 51 (18.6%)
Metabolic Syndrome	*n* = 43 (17.3%)
Noninvasive Measures	
LSM (*n* = 271)	Mean: 12.79 kPa ± 10.7; F1 = 69 (25.2%) F2 = 59 (21.5%) F3 = 44 (16.1%) F4 = 102 (37.5%)
CAP (dB/m) (*n* = 25)	223.04 ± 85.7
NAFLD fibrosis score (*n* = 271)	−1.27 ± 1.34
FIB4 (*n* = 271)	3.75 ± 12.2
HEPAMET score (*n* = 271)	0.12 ± 3.94
APRI (*n* = 271)	1.59 ± 3.94
FORNS (*n* = 271)	6.82 ± 1.95
Medical History (*n* = 271)	
Diabetes Mellitus	*n* = 36 (13.1%) − Diet *n* = 3 (1%) − OHAs *n* = 18 (6.5%) − Insulin *n* = 15 (5.4%)
Hypertension	*n* = 83 (30.2%)
Dislypemia	*n* = 45 (16.4%) – Diet 15 (5.4%) − Statins 10 (3.6%)
Psiquiatric Disorder	*n* = 55 (20%) – anxiety *n* = 45 (16.4%) – Psychotic disorder *n* = 10 (3.6% )
Toxics	Drugs *n* = 136 (49.6%); ExPWID *n* = 98 ( 35.7%); Tobacco *n* = 208 (75.9%)
Coffee	*n* = 173 (63.1%)
Child Pugh Score (A/B/C)	*n* = 92 (33.57%)/*n* = 10 (3.6%)/*n* = 0
Varices/Ascites/ Encephalopaty/HCC	*n* = 21 (7.7%)/*n* = 5 (1,8%) /*n* = 2 (0.7%)/ *n* = 3 (1.1)
Serum Biochemical Levels (*n* = 271)	
ALT, UI/mL	84.20 ± 66.97
AST, UI/mL	66.19 ± 51.725
GGT, UI/mL	88.28 ± 125.92
Bilirrubin, mg/dL	1.13 ± 4.51
Albumin, mg/dL	4.30 ± 0.34
Fasting Glucose Levels, mg/dL	92.96 ± 28.1
Insulin, µU/mL	11.11 ± 6.31
HOMA	2.56 ± 2.256
Platelets Count, 10*3/µL	174.12 ± 64.82
International normalized ratio (INR)	2.19 ± 10.13
Triglycerides, mg/dL	99.39 ± 45.38
Total Cholesterol, mg/dL	166.51 ± 39.12
High-Density Lipoprotein, mg/dL	51.34 ± 15.6
Low-Density Lipoprotein, mg/dL	96.44 ± 31.16

**Table 2:** Univariate and multivariate linear regression of Fibroscan^®^ values at 24 months. Univariate analysis showed significant logistic correlation between values of insulin, LDL-cholesterol, HOMA, and APRI. In multivariable analysis, the only one that maintained significance was HOMA. * *p* < 0.05.

	24M Elastography			
Variable	Pearson Correlation	*p* Value	Multivariate Regression	*p* Value
Insulin	0.356	0.0001*	−0.216	0.900
Cholesterol	−0.073	0.230		
HDL-Cholesterol	−0.028	0.652		
LDL-Colesterol	−0.122	0.050	0.265	0.125
HOMA	0.385	0.0001*	2.090	0.025*
Triglycerides	−0.009	0.886		
APRI	−0.165	0.006*	0.614	0.540
FIB4	−0.093	0.128	−0.111	0.903
Variable	OR (IC 95%)	*p* Value		
Metabolic Syndrome	5.4 (1.9–15.4)	0.001*		
BMI > 25kg/m^2^	(−0.656–3.423)	0.183		

## Supplementary Materials

The following are available online at https://www.mdpi.com/2077-0383/8/8/1242/s1, Supplementary File S1: Definitions and methods.

## Nonstandard Abbreviations Used

NAFLD, non alcoholic fatty liver disease; NASH, nonalcoholic steatohepatitis; ASH, alcoholic steatohepatitis; TIPS, transyugular intrahepatic portosystemic shunt; AST, aspartate aminotransferase; ALT, Alanine Aminotransferase; GGT, gamma-glutamil transferase; HOMA, homeostatic model assessment; LDL-cholesterol, Low-density lipoprotein cholesterol; HDL-cholesterol, High-density lipoprotein cholesterol; HCC, hepatocellular carcinoma; INR, international normalized ratio; APRI, AST to Platelet Ratio Index; FIB-4, Fibrosis-4 score; LSM, Liver-stiffness measurement; MELD score, Model of End-stage Liver Disease; CAP, controlled attenuation parameter; OHAs, oral antihyperglycemic agents.

## References

[1] European Association for the Study of the Liver (2018). EASL Recommendations on Treatment of Hepatitis C 2018. J. Hepatol..

[2] Garcia-Tsao G. (2018). Regression of HCV cirrhosis: Time will tell. Hepatology..

[3] Van der Meer A.J., Hansen B.E., Janssen H.L. (2013). Sustained virological response to treatment in patients with chronic hepatitis C—Reply. JAMA.

[4] Petta S., Di Marco V., Bruno S., Enea M., Calvaruso V., Boccaccio V., Rossi S., Ceaxi A., Camma C. (2016). Impact of virus eradication in patients with compensated hepatitis C virus-related cirrhosis: Competing risks and multistate model. Liver Int..

[5] Mandorfer M., Kozbial K., Schwabl P., Freissmuth C., Schwarzer R., Stern R., Chromy D., Stattermayer A.F., Reiberger T., Beinhardt S. (2016). Sustained virologic response to interferon-free therapies ameliorates HCV-induced portal hypertension. J. Hepatol..

[6] Perelló C., Cabezas J., Llop E., Carrion J.A., Ruiz-Antoran B., Llerena S., Crespo J., Calleja J.L., Hernandez-Conde M., Crespo J. (2017). LImpact of SVR in the development of all complications and fibrosis regression in a cohort of patients treated with interferon-base Triple Therapy and Direct Acting Antiviral. Hepatology.

[7] Ampuero J., Jimeno C., Quiles R., Rosales J.M., Llerena S., Palomo N.l., Cordero P., Serrano F.J., Urquijo J.J., Moreno-Planas J.M. (2018). Impact of comorbidities on patient outcomes after interferon-free therapy-induced viral eradication in hepatitis C. J. Hepatol..

[8] Hedenstierna M., Nangarhari A., El-Sabini A., Weiland O., Aleman S. (2018). Cirrhosis, high age and high body mass index are risk factors for persisting advanced fibrosis after sustained virological response in chronic hepatitis C. J. Viral Hepat..

[9] Rockey D. (2013). Translating an Understanding of the Pathogenesis of Hepatic Fibrosis to Novel Therapies. Clin. Gastroenterol. Hepatol..

[10] Pellicoro A., Ramachandran P., Iredale J.P., Fallowfield J.A. (2014). Liver fibrosis and repair: Immune regulation of wound healing in a solid organ. Nat. Rev. Immunol..

[11] Trautwein C., Friedman S., Schuppan D., Pinzani M. (2015). Hepatic fibrosis: Concept to treatment. J. Hepatol..

[12] Tacke F., Zimmermann H.W. (2014). Macrophage heterogeneity in liver injury and fibrosis. J. Hepatol..

[13] Fernandez M., Semela D., Bruix J., Colle I., Pinzani M., Bosch J. (2009). Angiogenesis in liver disease. J. Hepatol..

[14] Schuppan D., Kim Y. (2013). Evolving therapies for liver fibrosis. J. Clin. Invest..

[15] Grau-Bove X., Ruiz-Trillo I., Pascual F.R. (2015). Origin and evolution of lysyl oxidases. Sci. Rep..

[16] Puente A., Fortea J.I., Cabezas J., Arias Loste M.T., Iruzubieta P., Llerena S., Huelin P., Fabrega E., Crespo J. (2019). Loxl-2. A new target in antifibrogenic therapy?. Int. J. Mol. Sci..

[17] Wong C.C., Tse A.P., Huang Y.P., Zhu Y.T., Chiu D.K., Lai R.K., Au S.L., Kai A.K., Lee J.M., Wei L.L. (2014). Lysyl oxidase-like 2 is critical to tumor microenvironment and metastatic niche formation in hepatocellular carcinoma. Hepatology.

[18] Dongiovanni P., Meroni M., Baselli G.A., Bassani G.A., Rametta R., Pietrelli A., Maggoini M., Facciotti F., Trunzo V., Badiali S. (2017). Insulin resistance promotes Lysyl Oxidase Like 2 induction and fibrosis accumulation in nonalcoholic fatty liver disease. Clin. Sci. (Lond.).

[19] Bosch J., Ratziu V., Rockey D., Ghalib R., Thuluvath P., Schiefke I., Flamm S.,  Abdelmalek M., Millers R., Aguilar R. (2015). Ccorrelations between noninvasive markers of fibrosis and the hepatic venous pressure gradient (HVPG) in patients with compensated cirrhosis due to nonalcoholic steatohepatitis (NASH). Hepatology.

[20] Afdha P., Fortea J.I., Cabezas J., Loste M.T.A., Iruzubieta P., Llerena S., Huelin P., Fabrega E., Crespo J. (2015). Serum lysyl oxidase-like-2 (sLOXL2) is correlated with the hepatic venous pressure gradient (HVPG) in patients with cirrhosis due to hepatitis C. Hepatology.

[21] Bourliere M., Loustaud-Ratti V., Metivier S., Leroy V., Abergel A., Myers R., Aguilar R., Hyland R., Subramanian M., McHutchison J. (2015). Changes in liver stiffness by transient elastography (TE) and serum lysyl oxidase-like-2 (sLOXL2) in patients with cirrhosis treated with ledipasvir/sofosbuvir (LDV/ SOF)-based therapy. Hepatology.

[22] Barry-Hamilton V., Spangler R., Marshall D., McCauley S., Rodriguez H.M., Oyasu M., Mikekls A., Vaysberg M., Ghermazien H., Wai C. (2010). Allosteric inhibition of lysyl oxidase-like-2 impedes the development of a pathologic microenvironment. Nat. Med..

[23] Ikenaga N., Peng Z.W., Vaid K.A., Liu S.B., Yoshida S., Sverdlov D.Y., Mikels-Vigdal A., Smith V., Schuppan D., Popov Y.V. (2017). Selective targeting of lysyl oxidase-like 2 (LOXL2) suppresses hepatic fibrosis progression and accelerates its reversal. Gut.

[24] Loomba R., Lawitz E., Mantry P.S., Jayakumar S., Caldwell S.H., Arnold H., Diehl A.M., Djedjos C.S., Han L., Myers R.P. (2018). GS-US-384-1497 Investigators. The ASK1 inhibitor selonsertib in patients with nonalcoholic steatohepatitis: A randomized, phase 2 trial. Hepatology..

[25] Pollheimer M.J., Racedo S., Mikels-Vigdal A., Marshall D., Bowlus C., Lackner C., Madl T., Karlsen T.H., Hov J.R., Lyman S.K. (2018). Lysyl oxidase-like protein 2 (LOXL2) modulates barrier function in cholangiocytes in cholestasis. J. Hepatol..

[26] Tatal A.H., Feron-Rigodon M., Subramanian G.M., Bornstein J.D. (2013). Simtuzumab, an antifibrotic monoclonal antibody against lysyl oxidase like 2, appears safe and well tolerated in patients with liver disease of diverse etiology. J. Hepatol..

[27] D’Ambrosio R., Aghemo A., Fraquelli M., Rumi M.G., Donato M.F., Paradis V., Bedossa P., Colombo M. (2013). The diagnostic accuracy of Fibroscan for cirrhosis is influenced by liver morphometry in HCV patients with a sustained virological response. J. Hepatol..

[28] Lens S., Alvarado-Tapias E., Mariño Z., Londoño M.C., LLop E., Martinez J., Fortea J.I., Ibanea L., Ariza X., Baiges A. (2017). Effects of All-Oral Anti-Viral Therapy on HVPG and Systemic Hemodynamics in Patients With Hepatitis C Virus-Associated Cirrhosis. Gastroenterology.

[29] Puente Á., Cabezas J., López Arias M.J., Fortea J.I., Arias M.T., Estébanez Á., Casafont F., Fabrega E., Crespo J. (2017). Influence of sustained viral response on the regression of fibrosis and portal hypertension in cirrhotic HCV patients treated with antiviral triple therapy. Rev. Esp. Enferm. Dig..

[30] Fernández Carrillo C., Lens S., Llop E., Pascasio J.M., Crespo J., Arenas J., Fernandez I., Baliellas C., Carrion J.A., de la Mata M. (2017). Treatment of hepatitis C virus infection in patients with cirrhosis and predictive value of MELD: Analysis of data from the Hepa-C registry. Hepatology.

[31] Poynard T., McHutchison J., Manns M., Trepo C., Lindsay K., Goodman Z., Ling M.H., Albrecht J. (2002). Impact of pegylated interferon alfa-2b and ribavirin on liver fibrosis in patients with chronic hepatitis C. Gastroenterology.

[32] Llerena S., Perello C., Hernandez M., Garcia M., Ramos D., Estebanez A., Cabezas J., Cuadrado A., Lopez Hoyos M. (2016). Endothelial dysfunction, macrophage dysfunction and emerging cardiovascular risk factors in patients with hepatitis c virus infection. characterization and potential reversibility with direct acting antiviral agents. J. Hepatol..

