# Supplementary material for: Changes in Circulating Lysyl Oxidase-Like-2 (LOXL2) Levels, HOMA, and Fibrosis after Sustained Virological Response by Direct Antiviral Therapy
Source: J Clin Med. 2019 Aug 17;8(8):1242. doi: 10.3390/jcm8081242 (PMC6723423; doi:10.3390/jcm8081242)
Supplement: Supplementary file 1 [file jcm-08-01242-s001.pdf]

Influence of the metabolic syndrome on fibrosis regression regulated by LOXL-2 after sustained virologycal response.

Supplementary information 1.

Definitions

Sustained virological response: The decision of treatment was at discretion of the treating physician, following the current recommendations of EASL Guidelines and Spanish Association for the Study of the Liver. SVR was defined as an undetectable HCV-RNA 12 and 24 weeks after the end of treatment. Virological failure was considered if HCV-RNA became detectable during treatment or post-treatment follow-up. HCV-RNA levels were determined using either the COBAS AmpliPrep/COBAS TaqMan (Roche Molecular Systems, Pleasanton, CA; lower limit of detection, 15 IU/mL)

Genotyping/subtyping: were performed with a method based on reverse hybridization with the line probe assay.

Metabolic syndrome (Mets) MetS was defined as the fulfilment of at least three of the following criteria: (a) abdominal obesity (waist: males ≥ 90 cm, females ≥ 80 cm); (b) blood pressure (systolic pressure [SBP] ≥ 130 mm Hg or diastolic pressure [DBP] ≥85 mm Hg); (c) hyperglycemia (fasting glucose [FG] ≥ 100 mg/dL); (d) HDL-C (males < 40 mg/dL, females <50 mg/dL); and (e) TG ≥150 mg/dL, according to the Joint Scientific Statement "Harmonizing the Metabolic Syndrome."

Liver steatosis: The presence of steatosis will be defined as the presence of at least one of the following. -CAP> 288 dB/m, or FLI index> 60.

Significant liver fibrosis by noninvasive methods: LSM >9kPa, NAFLD fibrosis score > 0.675 Forns > 6.9 .APRI >1.5 and FIB 4 >3.25

HVPG, liver biopsy and LSM

Hemodynamic studies were performed after overnight fasting under local anesthesia, a venous introducer was placed in the right internal jugular vein using the Seldinger technique. Under fluoroscopy, a 7F balloon-tipped catheter (Edwards Lifesciences, Irvine, CA) was guided into the main right hepatic vein for measurements of wedged hepatic venous pressure and free hepatic venous pressure. The adequate occlusion of the hepatic vein was checked by manual injection of a small amount of radiologic contrast medium. Free hepatic venous pressure was measured in the right hepatic vein close to the inferior vena cava. The portal pressure gradient was measured as HVPG (the difference between wedged hepatic venous pressure and free hepatic venous pressure).

Transjugular hepatic biopsy was performed during the portal hemodynamic procedure. Under radiological control using the Tru-Cut technique, at least two liver samples of 1.5cm in length were obtained. One of them will be sent to the Pathology Service for conventional study. Hepatic fibrosis was quantified by Masson's trichrome staining and

collagen quantification (I, III and IV). The inflammatory infiltrate and fatty deposit by hematoxylin-eosin. A second sample was processed in the laboratory of our group, being frozen for the first time at -80 ° in RNA stabilizing medium (RNAlater ®). These samples will be directed to perform different studies of gene and protein expression of LOXL2. The expression analysis of LOXL2 will be carried out through mRNA expression arrays (Human GE 8x60K Microarray, Agilent, Affymetrix eBioscience). The total RNA will be prepared using the Trizol reagent (Invitrogen, Carlsbad, CA, USA) and its quality will be analyzed by means of a total RNA (small fraction chip) coupled to a Bioanalyzer 2100 (Agilen Technologies Inc., Santa Clara, CA) and by electrophoresis in agarose gel. The results will be validated in a second time by RT-qPCR.

Measurement of liver stiffness (LSM) was performed by transient elastography (Fibroscan®; Echosens, Paris, France), under the usual quality criteria at baseline and 24 months after antiviral treatment. All procedures were made by M probe. Cutoff values: F1 ≥5.3 kPa; F2≥ 7.4kPa; F3 9.0 kPa; F4 ≥13.2 kPa.

Serum Loxl2:

At baseline and 24M, we obtained LOXL2 through ELISA technique (Human lysyl oxidase-like 2 ELISA Kit MBS904757. Biosource

Statistical analysis.

A descriptive analysis was performed. Categorical variables were described with percentages, and continuous variables were described with mean and standard deviation or median and range/interquartile range (IQR), as appropriate. A 95%CI was considered to estimate proportions. The statistical analysis was performed with SPSS Statistics for Windows, Version 21.0 (IBM Corp, Chicago, Armonk, NY, USA). All p-values were two-tailed and statistical significance was defined as $p<0.05$.

The Kolmogorov–Smirnoff test was used to check the normality of the data distribution. Mean values were compared among groups with the 1-way analysis of variance (ANOVA), followed by the Tukey multiple-comparison test, the unpaired Student 2-tailed t test, or nonparametric Mann–Whitney U test, as appropriate. Correlations were examined by Pearson standard linear regression analysis (normal distribution) or by the Spearman test (non normal distribution).
